# What is appropriate care? A qualitative study into the perceptions of healthcare professionals in Flemish university hospital intensive care units

**DOI:** 10.1016/j.heliyon.2023.e13471

**Published:** 2023-02-03

**Authors:** Lore Huwel, Joke Van Eessen, Jan Gunst, Manu L.N.G. Malbrain, Veerle Bosschem, Tom Vanacker, Sofie Verhaeghe, Dominique D. Benoit

**Affiliations:** a Ghent University Hospital, Department of Intensive Care Medicine, Corneel Heymanslaan 10, 9000 Ghent, Belgium; b Leuven University Hospital, Department of Intensive Care Medicine; Campus Gasthuisberg, Herestraat 49, 3000 Leuven, Belgium; c KU Leuven, Department of Cellular and Molecular Medicine, Laboratory of Intensive Care Medicine, Onderwijs & Navorsing 1 (O&N1) Building of Campus Gasthuisberg, Herestraat 49, 3000 Leuven, Belgium; d Brussels University Hospital, Department of Intensive Care; Brussels Health Campus, Laarbeeklaan 101, 1090 Jette, Belgium; e International Fluid Academy, iMERiT vzw, Dreef 3, 3360 Lovenjoel, Belgium; f Ghent University, Centre for Nursing and Midwifery, Department of Public Health and Primary Care, UZ Gent, 5K3 (entrance 42), Corneel Heymanslaan 10, 9000 Gent, Belgium; g VIVES University College Leuven, Department of Nursing, VIVES Roeselare, Wilgenstraat 32, 8800 Roeselare, Belgium; h Hasselt University, Faculty of Medicine and Life Science; Agoralaan, 3590 Diepenbeek, Belgium; i Department of Intensive Care Medicine, Ghent University Hospital, Corneel Heymanslaan 10, 9000 Ghent, Belgium

**Keywords:** Qualitative research, Appropriate care, Intensive care, Interdisciplinary collaboration, Decision-making climate

## Abstract

**Aim:**

This study examines when healthcare professionals consider intensive care as appropriate care.

**Background:**

Despite attempts to conceptualize appropriate care in prior research, there is a lack of insight into its meaning and implementation in practice. This is an important issue because healthcare professionals as well as patients and relatives report inappropriate care in the intensive care unit (ICU) on a regular basis.

**Methods:**

A qualitative study was designed, based on principles of grounded theory. Seventeen semi-structured interviews were conducted with nurses, doctors and doctors in training from three Flemish university hospitals. Analyses followed the Quagol method; insights were gained by means of the constant comparative method.

**Results:**

Healthcare professionals described appropriate care as socially sustainable care, high-quality care, patient-oriented care, dignified care and meaningful care. They considered it important that care is not only proportional to the expected survival and quality of life of the patient and in line with the patient's or relatives’ wishes, but also that the pursuit of the care goals is proportional to the patient's suffering.

Although healthcare professionals indicated the same elements of appropriate care, they were defined and interpreted in individual and therefore different ways. This diversity lies at the basis of fields of tension and frustrations among healthcare professionals.

**Conclusion:**

Appropriate care is defined and interpreted in individual and therefore different ways. In order to decide which type of care is appropriate for a specific patient, a process of open and constructive communication in a team is recommended.

## Highlights

• Equal distribution of care and sustainability of the healthcare system are crucial.
• The intensity of care must be proportional to an individual's estimated prognosis.
• The pursuit of future prospects is proportional to the patient's suffering.
• Integrating the perspectives of doctors and nurses can reduce uncertainty.
• A framework could facilitate reflecting on appropriate care in an ICU team.

## 1 Introduction

In a rapidly changing society, health organizations are faced with various trends which challenge sustained high quality, affordable care [1]. These include, for example, the continuous technological and scientific evolution, an ageing population, polycomorbidities in patients and savings in the health sector [2,3]. Healthcare providers are increasingly confronted with ethical dilemmas, such as withholding and withdrawing treatments, and allocating resources [4,5]. For this reason the World Health Organization considers making sound choices in the patient care process a challenge and their main objective [6].

The complex character of the concept of appropriate care is illustrated by the nuanced definitions used in different studies [7,8]. Definitions often describe conditions for the term appropriate care, such as cost-effectiveness and respecting the main ethical principles [7,9]. Other aspects of appropriate care include evidence-based care, clinical expertise, patient-centered care, use of resources, and fairness [2,8,10].

An unambiguous definition of appropriate care is lacking [11]. Appropriate care is linked to the quality of care. Indeed, providing appropriate care results in optimal, high-quality holistic healthcare [12]. In the context of high-quality care, efforts have been made worldwide in recent years to develop clinical guidelines for appropriate care in accordance with international best practices [2].

Research within intensive care unit (ICU) communities shows that despite these efforts, such guidelines do not always lead to administered care being perceived as appropriate by healthcare professionals, patients and family members [8,11,[13], [14], [15], [16], [17], [18]]. ICU healthcare professionals signal care which is no longer proportional to the expected prognosis of the critically ill patient on a daily basis [13,16]. Moreover, such perceptions of inappropriate care by care providers appear to be strongly predictive for patients' chances of survival and their quality of life [16].

A possible explanation for the discrepancy between the care prescribed by the guidelines and the care a patient effectively receives is the fact that the clinical guidelines are not adapted to the local contexts of practice [7,19]. In addition, Robertson-Preidler et al. [10] report that having insight in the concept of appropriate care through research is an important condition before it can be implemented in practice.

In view of the lack of a formal definition of appropriate care and the fact that cases of inappropriate care are still being reported, this study aims to increase insight in the concept of appropriate care. This may reveal reasons why the daily provision of appropriate care in the ICU does not always seem to be obvious [20]. This study attempts to (1) map the views of nurses, doctors and doctors in training, feeding a definition of appropriate care provision in the local context of ICU and (2) identify the principles and concepts that emerge from their views about appropriate care provision. These new insights can contribute to the scientifically based pursuit of an ethical working climate in which appropriate care is optimized.

## 2 Methods

This is a qualitative study using interviews with clinical ICU staff that were analyzed using the Quagol method, a practice-based guide that is inspired by the constant comparative method of the Grounded Theory Approach [21]. This study was approved by the ethics committees of all participating centers (Belgian registration number B670201734533).

### 2.1 Participant recruitment and data collection

In the period between April 2018 and February 2019, nurses, doctors and doctors in training, working on an intensive care unit in a Flemish university hospital were invited to participate in the study. Healthcare professionals with at least three months' work experience in an adult surgical or medical ICU were included in a purposive sample through contact persons in the centers. Healthcare professionals solely working in a cardiosurgical ICU were not eligible. Healthcare professionals were initially included based on their interest in participating in the study. As the study progressed, the contact persons were asked to recruit healthcare professionals on a more targeted basis in terms of properties of gender, position, age and years of work experience at an ICU. In this way we attempted to find and monitor a balance between homogeneity and heterogeneity of the sample.

The participants were invited to a one-time interview. The interviews started with the open question when care is considered as appropriate care by the participant. Guided by the answers of the participants, in-depth questions were used to assess practical examples and associated emotions and experiences. During the interviews, field notes were taken of remarkable situations and leads for further inquiries. As the interviews went on and more respondents joined the study, common themes that needed further clarification were used in the creation of follow-up questions (Table 1). Some sociodemographic data were queried via a structured questionnaire. An interview lasted an average of 46 min and took place at the participant's home or at the workplace, depending on the participant's preference. After obtaining the staff member's consent, the interviews were recorded on an audio tape and afterwards fully and anonymously transcribed with attention to verbal and non-verbal communication.

**Table 1:** Guiding questions and theme-based questions from the interview guide.

**Opening question**
When do you consider care as appropriate care?
**Follow-up questions**
How would you describe appropriate care?
You talked about … Could you tell something more about it?
Can you give a specific example?
How did you feel about that situation?
What did it mean to you?
How did you cope with it?
If you had the authority to do it, how would you do it?
**Follow-up questions based on themes that need further declaration**
What do you mean with patient-centered?
How do you see care that is socially sustainable?
What makes it difficult to express your point of view?

### 2.2 Data analysis

The interviews were analyzed using the Quagol method. The method is characterized by iterative processes with forward-and-backward dynamics that facilitate the formulation of concepts and categories to build the structure of the research answer [22].

Four phases of data collection and data analysis succeeded each other in a cyclical process. After a phase of data collection with two to six participants, a phase of systematic analysis of the obtained data followed.

In a first step, each participant's storyline was articulated in the light of the research question. Subsequently, this storyline was clustered into a number of categories, characteristic concepts and theoretical memos that acted as preliminary response to the research question (preliminary hypotheses).

On the basis of the insights gained by the researcher, new participants were queried in a targeted manner, so that their data could be tested against the formulated preliminary hypotheses. In this way, the focus was progressively on obtaining in-depth data. Common categories and concepts were described in an overarching conceptual interview scheme that served as the basis for designing a code tree in the NVIVO 12 Pro software package. This code tree was adapted and refined through the assignment of codes to the data. Each new code was verified in the light of the previous interviews. Guided by the theoretical memos and analysis of the codes, categories and concepts emerged more clearly. Throughout the entire study, data collection and analysis focused on linking concepts and formulating a clear answer to the research question. Participant inclusion was terminated when the obtained data no longer yielded new insights and when the researcher's interpretive ideas appeared clear.

### 2.3 Research quality

An audit trail tracked the researcher's decision-making process to ensure the transparency and accuracy of the investigation [21]. In order to avoid subjectivity in the data collection and data analysis, the researcher drew up a frame of reference with which an attempt was made to formulate an answer to the research question based on her own assumptions. In addition, through peer review and research triangulation, intensive consultation was conducted with the research group about all steps in the study in order to be able to detect bias and inappropriate subjectivity, to test alternative explanations of data and to maximize the validity of the interpretations [23].

## 3 Results

### 3.1 Properties of the participants

Seventeen individual, semi-structured interviews were conducted with nurses, doctors and doctors in training from three Flemish university hospitals. All three hospitals are considered tertiary referral hospitals offering the complete suite of specialized medical care and expertise. Their core goals are innovation, education and patient-centered care. Table 2 shows an overview of the properties of the participants.

**Table 2:** Properties of the participants (N = 17).

	Position			
	Nurse	Doctor	Doctor in training	*Total*
Sex				
Man	2	4	0	*6*
Woman	7	2	2	*11*
*Total*	*9*	*6*	*2*	*17*
Age (years)				
21-30	2	1	1	*4*
31-40	2	2	1	*5*
41-50	3	3	0	*6*
>51	2	0	0	*2*
*Total*	*9*	*6*	*2*	*17*
Work experience in the ICU (years)				
0-5	2	2	2	*6*
6-10	1	1	0	*2*
11-20	2	3	0	*5*
21-30	4	0	0	*4*
*Total*	***9***	***6***	***2***	*17*

### 3.2 Subjective interpretation of appropriate care

Describing when intensive care is considered as appropriate care proved difficult and extensive for each participant (Table 3, Q1). In addition, the participants judged that appropriate care may imply different things in each individual patient case, depending on the patient's health status, needs and preferences (Table 3, Q2). During the interviews, the participants initially tried to look for elements that could be part of a transcending, commonly supported definition of appropriate care. The participants used concepts that appeared to be defined unambiguously, such as correct care, respectful care, dignified care, patient-oriented care and socially sustainable care (Table 3, Q3, Q4, Q5, Q6). However, when the healthcare professionals translated the concepts they had mentioned into concrete patient cases, they no longer appeared to have such unambiguous reference. They then reverted to their own subjective conceptual frameworks to interpret the concepts in concrete ways. They frequently related their assessments of appropriate care to the impressions and emotions gained in the patient case and found it easier to indicate what they considered inappropriate, rather than what type of care they considered appropriate (Table 3, Q7).

**Table 3:** Illustrative quotes indicating the subjective interpretation of appropriate care^2^.

Quote number	Quote
Q1	(Doctor) “… I find that a very difficult question and I don't have a ready answer to it because I think it is very, very difficult to put that into a general definition. (…) I think that is a very broad concept that actually encompasses a lot of things like a very large umbrella …”
Q2	(Doctor) “… But that is, that is infinitely diverse. I think, yes, you have five patients, that's five different appropriate cares I think, that you can have. …”
Q3	(Doctor) “… Appropriate care towards the patient, in which your patient receives appropriate care, in which he actually receives the care he should receive, which is therefore evidence based. With an assurance of quality, yes, something like that. (…) Yes, then you also have the financial aspect, financially appropriate. …”
Q4	(Nurse) “… Generally, that the care is appropriate, that it is also, um, what is very important to me is that it remains ethically appropriate. (…) Um, that there is not much overtreatment … “
Q5	(Nurse) “… We must, of course, always respect the patient and, well, it is a human being that you want to treat with dignity. Um, but yes, that is sometimes very difficult, that we have to continue the therapy like this, again and again. To give substance to that meaninglessness, for us, so um, the meaninglessness of care is often a difficult point for us. …”
Q6	(Nurse) “… For myself, that is appropriate care, that I remain strict with myself and that I continue to force myself to look it up or check it out or ask a colleague. Actually questionning yourself a lot, whether you are still providing appropriate care, and is this up to date or is this correct. …”
Q7	(Nurse) “… Um, appropriate care, yes, I think the outcome is especially important. And indeed, you have to comply with the principles, the protocols, as best as possible, that can differ sometimes … (…) Those protocols are also there to indicate a correct process and that, I think, also leads to appropriate care.” Interviewer: “Can you give an example?” Participant: “(…) When you see the patient is suffering and the family is suffering, then you're not going to wait for the doctor to finally put that prescription in to start [starting up morphine injection]. That makes it humane and I also think that is appropriate care.” Interviewer: “(…) That humane aspect, how do you determine that?” Participant: “Intuition I guess. I think everyone has to decide that for themselves. It's hard to put into words, but yes. Human dignity is something, I think that it is something you stand for and I think that is also somewhat determined by the ward you work for. I think we all have the same visions here about what is humane and what is not. But yes, I find that difficult. (…) We are experiencing a lot of misery here. Um, and yet you want people to die, live and be treated with dignity. Um, yes, but how you determine whether something is humane, I think is ethics, isn't it. That is, there are also a lot of rules for ethics. …”
Q8	(Doctor in training) “… I just see that in colleagues, I see that in staff members, I see that in various disciplines. You have people who are extremely focused on the psychological aspect, you have people who are extremely focused on numbers, and you have people who are extremely focused on the theory, and you have people who are extremely focused on all the tubes coming out of the patient. And depending on their focus, their appropriate care is going to be everything has to be sterile, everything has to … Yes, that differs. …”
Q9	(Nurse) “… But that depends a lot on which doctor that is on duty, you have doctors to whom everybody would say anything and then others if it is him, then I won't open my mouth because he talks us down anyway. So, yes, it very much depends on the person. (…) Yes, you have some who really listen like "oh, is this true? Tell me or what do you mean? "And you have others who show less understanding. You are supposedly wrong and that cannot be the case and on paper it is all okay. Go. Actually blocking you …”

During the interviews it also emerged that in practice it often proved to be difficult to reach a consensus within the care team about the appropriate treatment for a specific patient. Although healthcare professionals seemed to use the same concepts, they were defined and interpreted in individual and therefore different ways (Table 3, Q8). In order to achieve consensus, a uniform framework for assigning weights to everyone's arguments seemed to be lacking (Table 3, Q9).

### 3.3 The five elements of appropriate care

**Fig. 1:**
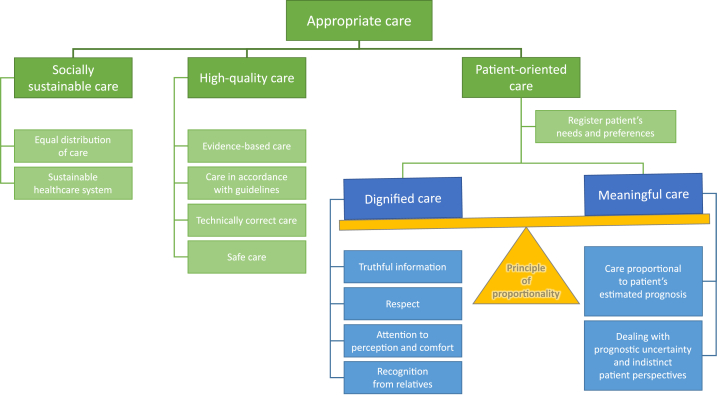
The five elements of appropriate care.

In an attempt to clarify the confusion of concepts and in order to be able to discuss the results with the unified terminology, we tried to draw up a conceptual framework (Fig. 1). Five elements of appropriate care emerged systematically from the data. An attempt was made to interpret their potential meanings and relationship within this framework. The structure is inevitably artificial, as the boundaries between the various aspects cannot always be clearly defined.

The conceptual framework was mainly formed on the basis of the perceptions of four self-reflective participants. These healthcare professionals were able to identify core elements and conditions for themselves in order to speak of appropriate care. For the other participants it seemed more difficult to do this exercise. It was mainly when talking about concrete patient cases that it became clear what meaning certain concepts seemed to have for them. Although the classification of concepts was not made so explicit by them, their visions could be mapped to the conceptual framework.

The elements "socially sustainable care " and "high-quality care" proved to be the most unambiguous concepts for the participants. The element of "patient-oriented care" was the most difficult to describe and turned out to be interpreted in very individual ways. An attempt was made to further categorize this aspect in line with what participants potentially perceived as patient-oriented care.

#### 3.3.1 Socially sustainable care

Within "socially sustainable care" the principles of equal distribution of care and sustainability of the healthcare system are central (Table 4, Q1). For example, overconsumption, in particular the prescription of (expensive) examinations and/or treatments without any indication, was not justified according to the participants and should therefore be rejected (Table 4, Q2). In addition, they considered it the responsibility of the healthcare team to ensure the balance between the use of invasive, highly specialized treatment techniques and the benefits for patients. However, the data showed that the perceived pressure to watch over this balance on a daily basis seemed to be much smaller than the experienced fear of making mistakes, being negligent and running legal risks (Table 4, Q3).

**Table 4:** Illustrative quotes concerning the five elements of appropriate care^3^.

Element of appropriate care	Quote number	Quote
1. Socially sustainable care	Q1	(Doctor) “… On the other hand, it is something that should be fair or equitable, everyone should be given that chance. If there's something that can be done, but we can only give it to one person in a hundred, but all a hundred need it, then you're in some sort of conflict. (…) If you talk about a device that can help or a certain chemotherapy, it must be feasible for our society to support it financially. (…) We are lucky that we don't have to look at the bills, but sometimes we should stop to think about it. Hundred days of intensive care costs a lot of money and we don't think about it enough. …”
	Q2	(Doctor) “… But I think you also have to take society into account because you are ultimately working with taxpayers’ money as well. Um, giving extremely expensive therapies with very little chance of success to people who already have very little quality of life beforehand, I think is totally inappropriate. …”
	Q3	(Doctor in training): “… You actually want to cover yourself, you want a lot of checks. Because you think “oh, intensive care”. You want a lot of checks. You don't want to miss anything. …”
2. High-quality care	Q4	(Doctor) “… Appropriate care is where you make sure that you provide correct care to your patient, that there is no negligence and that you can say with good conscience that “the best possible thing happened here”, even if it is not always the best decision taken at. …”
	Q5	(Nurse) “… Based on yes, the most recent research and studies and views. And remaining open to new insights. …”
	Q6	(Doctor in training) “… That safety, I check it. The security that I know right now. I'm sure there are some things I'm overlooking right now. (…) But the things that I know now must be correct. I simply insist on that. …”
3. Patient-oriented care	Q7	(Doctor) “… But besides what we think we should give, we have what the patient expects, um, as maybe someone uninformed medically, but the other looking at their life, certainly in the ICU. (…) It may not be the care that is purely patient-driven, but the outcome that is patient-driven, in the sentence "I find this acceptable". Um, what you find acceptable as a 50-year-old is probably different from what is acceptable to you as an 80-year-old. …”
4. Dignified care	Q8	(Nurse) “… And especially let patients participate in the decision-making process, involve them in how good or bad it is now. It may be, yes, he is always entitled to say yes or no in therapy. To co-decide. …”
	Q9	(Doctor in training) “… Um, and then, respect for the body. Yes, try to respect privacy as much as possible. So yeah, I just pay attention to that in my daily practice. I am really attentive to that. I try to do that as best I can. …”
	Q10	(Nurse) “… For me, appropriate care means that your patient has the necessary comfort, taking this into account. That's really important. What I see is yes, that there is a lot of dragging on people. And then I ask myself whether that is still necessary sometimes. I have a hard time with that. The sick are much sicker than twenty years ago, at least that's what I think. …”
	Q11	(Nurse) “… But not only correct care, it's not just about putting the right baxter on here, give the right pills here, but also about making time for the patient. So, and that is something that often is … But that is appropriate care for me, so that is also the thing for me, giving the right medication, but also that the patient, yes, also gets the care on an emotional level that he or she needs. …”
	Q12	(Nurse) “… But what is also an incidental thing for me, is that appropriate care is actually also a bit the care, not of the patient when he is sedated, but of the family. That is also appropriate care for me. (…) Often I will stay with the family longer because then you sometimes feel that they need to ask the nurse something more because the nurse is easier to reach, so to speak. (…) But then I try to be present for that family; so that they get the feeling like ‘ok; if we want to ask something’ … “
5. Meaningful care	Q13	(Doctor) “… And I think that is also part of appropriate care where you do not always do everything for the people who have to get all that, but sometimes we also say that we are moving towards a reduction of therapy or a limitation of therapy. Because you can take in anyone, you can give everyone organ replacement therapy, dialysis, ventilate, you can keep someone alive for a very long time, but I think you are not going to render a service to society and people with that. So I think so. …”
	Q14	(Nurse) “… But that shouldn't be the goal, doing just that, we'll take them upstairs [the nursing ward]. No, you also have to see the future, right. It's not because you put them upstairs that you have given good care, I think. Away from intensive and then they are cured, no, then it just starts. …”
	Q15	(Doctor in training) “… But actually mainly looking at what is best for the patient, in a patient-oriented way. And also being realistic in what you still do and what you don't do. I think that sometimes it goes a little too far here, that we are sometimes too persistent in our therapies. And that we should especially look at what is the patient like, in what state are they, in what condition are they now, what were they like beforehand, how are they surrounded socially, these are all important aspects. … “
	Q16	(Nurse) “… You should always find out about this with the relatives, with loved ones, like has he already talked about it in the past, or she already talked about it, would he want that. Of course it is always a guess, but that way we can gauge more or less what he would have wanted this way or that way. But yes, that is, um, slippery ice, right, to decide what is quality and what is not. … “
	Q17	(Doctor) “… It's not that easy sometimes, right, to make a decision in some cases. Uh, and yes, you can … If you really say to the end-of-life, that's something you can only take once. So and, yes, we have to make that decision. … “
	Q18	(Doctor) “… It is actually about the whole sphere of life in which that patient, in which that person who has become a patient, actually lives. With everything that is important to that patient. And if you can integrate that into fitting care, I think you will then have appropriate care. (…) … I think that the GP has an important role to paint a picture of the patient and I think that we sometimes do not involve them enough. We start from scratch. We don't know that context. And sometimes when you call the GP, you get a completely different view, and that is very important, I think, to be able to frame your care. …”
	Q19	(Nurse) “… Euhm, that is respecting, uh, the person lying there in that bed, euhm, seeing them as a person and above all asking the question" would I want to lie here like that? Would they, now suppose I lie in that bed in a similar situation, would I want them to do all that to me?" … "
*Principle of proportionality between dignified care and meaningful care*	Q20	(Doctor) “… If you want to torture someone, the ICU is one of the best places to get patients to undergo it. So you have to take that into account. So how I look at that, indeed, what do you still want to do for your patient, you want to save your patient, but then again, of course, you can't make the patient suffer either. And, and everyone has sort of their own opinion and their own vision on that. … “
	Q21	(Nurse) “… Yes, we often give the example like yes, you see them a few times a day, we see them all the time. (…) While we see them day and night, you have to take care of them, you have to hurt them. Yes. And they don't really see that. (…) Yes, then they say "it is going well, isn't it?", "It works, right?", while that is not always the case of course. …”
	Q22	(Doctor) “… Often it is. You see little things, but those little things are surrogate makers. And yes, we have reduced some oxygen, we are in a phase with less sedation, but objectively you are still very far away. Um, and that may be a habit of how I grew up in the hospital. Once you say A, you also want to do B and C and you think, I have to do my homework well, I have to give that person a lot of opportunities and I will start with that and we have kidney replacement therapy and we are going to place a tracheo anyway. Yes, and if you have placed the tracheo, yes, you still have to take two more weeks to wean off the ventilator. While in the meantime things sometimes happen that show that the chances remain very low or have even decreased somewhat. … “

#### 3.3.2 High-quality care

Participants believe that the entire healthcare team should have expertise in identifying treatment options and delivering care. Healthcare professionals should be able to provide the best possible care in good faith, without being negligent (Table 4, Q4). "High-quality care" is defined as being in line with current scientific insights and guidelines for care (Table 4, Q5). Techniques and procedures must also be performed correctly, guaranteeing patient safety (Table 4, Q6).

#### 3.3.3 Patient-oriented care

The element of "patient-oriented care" was the most difficult to describe. The healthcare professionals agreed on three central principles, but all healthcare professionals seemed to interpret those principles in very individual ways, based on their personal values and norms, knowledge, experiences and responsibilities.

The first central principle is that care must be patient-oriented. Healthcare professionals interpreted this in terms of care in accordance with the needs and preferences of the patient (Table 4, Q7). To this end, care goals should be formulated in an open and honest dialogue with the patient.

#### 3.3.4 Dignified care

The second central principle is that the patient must be treated respectfully. The participants associated this with various universally applicable principles, such as the right to truthful and complete information, respect for the patient, monitoring the patient's comfort and experience, and attention to relatives and loved ones (dignified care; Table 4, Q8, Q9, Q10, Q11, Q12).

#### 3.3.5 Meaningful care

As a final central principle, healthcare professionals considered it the doctor's responsibility to ensure that the intensity of care is proportional to an individual's estimated prognosis (meaningful care; Table 4, Q13). All healthcare professionals unanimously stated that it is justifiable to the patient, their relatives and the healthcare team to pursue maximum chances of survival for the patient. Nevertheless, the pursuit of survival should not be an isolated goal in their view. From an ethical point of view, participants said it is also important to consider both survival and estimated quality of life for the patient after admission to ICU when making treatment decisions (Table 4, Q14). What quality of life entails for someone seemed to be highly individually determined according to the participants. Moreover, this can also change once one is confronted with reduced functionality, they stated. In determining meaningful treatment options, it, therefore, seemed important to map out the patient's needs and preferences, in addition to the survival chances and the estimated quality of life after ICU (Table 4, Q15).

One challenge that healthcare professionals raised in the interviews is dealing with prognostic uncertainty and uncertainty about the possible impact of patients' health status on their quality of life. An additional barrier that healthcare professionals often face in the ICU lies in the fact that the patients' views cannot be questioned directly because their health status does not allow this. This lack of clarity about a patient's perspective increases uncertainty about the decisions to be taken even more (Table 4, Q16). Both challenges must be addressed in order to determine treatment options that are commensurate with the patient's expected prognosis and quality of life. The existence of fear for the consequences of treatment decisions made by healthcare professionals is inherent to these (subjective) assessments (Table 4, Q17).

The data showed that doctors consulted different sources to gain insight in the patient's perspective. In dialogue with other healthcare professionals, the patient's relatives and GP, various types of information were mapped for this purpose, such as data about the entire life sphere, the social network and the patient's personal beliefs and values (Table 4, Q18). To counter uncertainty about the patient's future prospects, doctors found it valuable to plan interim care goals and make interim assessments of the patient's health status. The interviews showed that nurses also assessed the meaningfulness of treatment decisions, even though they do not bear final responsibility in the decision-making. However, the nurses used a different frame of mind. They mainly judged on the basis of what they themselves would like if they were in the same situation as the patient (Table 4, Q19). As a result, they attached great importance to personal values and norms, personal life experience and confrontation with suffering during their professional careers, success stories, powerlessness and insecurity in their definitions of meaningful care.

#### 3.3.6 Principle of proportionality between dignified care and meaningful care

The data showed that the participants did not only consider it important that the intensity of care is proportional to an individual's estimated prognosis (dignified care), but also that the pursuit of future prospects for the patient is proportional to the patient's suffering (meaningful care). The principle of proportionality is double sided in nature (Table 4, Q20).

**Fig. 2:**
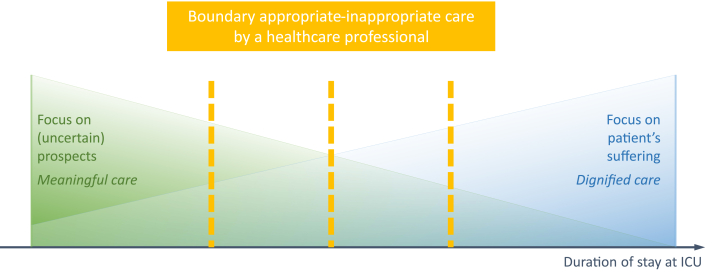
Diversity in the opinion of healthcare professionals about proportionality between meaningful care and dignified care.

Where the boundary between appropriate and inappropriate care is situated in this field of tension between dignified and meaningful care, seemed to be determined on a very individual basis by each clinical staff member (Fig. 2).

When the staff member's motivation in a specific patient case was mainly focused on pursuing the patient's comfort (focus on dignified care), it was possible that staff member's boundary was reached early in the care process and at the least suffering of the patient. By contrast, it was only possible for a clinical staff member who mainly had the (uncertain) life chances of the patient in mind (focus on meaningful care) to conclude later in the care process that continuation of therapy was no longer useful. The interviews showed that clinical staff members did not find one motivation more correct than the other. In addition, a preference to strive for comfort did not preclude striving for progress, or vice versa. The staff members' personal motivation seemed to determine how they perceived the patient's suffering and the progress in the patient's condition. The data showed that the closer the clinical staff member was to the patient's bedside and thus the more intensive the contact moments with that patient were, the more the patient's suffering weighed in the assessment of proportionality between dignified and meaningful care. In such cases the patient's suffering could become so prominent in a staff member that small improvements in the patient's condition were hardly seen as progression. Strengthened by professional experience and confrontation with suffering, clinical staff members could then make the subjective assessment that the patient's palpable suffering was disproportional to the small chances of survival (Table 4, Q21). By contrast, the absolute pursuit of relevant progress in the patient's health status could imply that the patient's suffering is acknowledged insufficiently (Table 4, Q22).

### 3.4 Integration: field of tension among healthcare professionals regarding appropriate care

When incapacitated patients had not been able to express their preferences in advance and when there was great uncertainty about the patients' future prospects, clinical staff members sometimes had different views regarding the type of care which was appropriate for those patients. Based on this diversity in the perspectives, a field of tension could arise among healthcare professionals (Table 5, Q1). When clinical staff members were forced to continue cooperation to a potentially curative treatment without taking into account the patient's underlying suffering, they could become frustrated. The interviews showed that nurses faced doubts about the proportionality between dignified and meaningful care first more often than doctors.

**Table 5:** Illustrating quotes indicating the field of tension among healthcare professionals regarding appropriate care^4^.

Quote number	Quote
Q1	(Nurse) “… It's more difficult when those patients have been there for a long time and we don't see any improvement and the doctors are sometimes vague. Of course it is sometimes difficult because it is multidisciplinary. Surgeons, usually even different surgeons, our doctors who also have their own opinions among themselves. Euhm, and then adding our opinion and your own vision behind it. It is sometimes difficult to get all of that aligned. And then it is more difficult in such cases indeed to call it appropriate. … “
Q2	(Doctor) “… We are sometimes turned into our own thing. An oncologist goes very far with his thing, a cardio surgeon goes very far with his thing. And we have that too. We claim not and we say 'it is the surgeon.' We sometimes continue for several weeks and sometimes you have to look objectively from the outside. And that is difficult. And when you switch supervision, you sometimes get that. You have taken care of a patient for three weeks, you are in a certain frame of mind and you are taking over from me, you have not followed those three weeks and you look objectively at that man, that patient, and say 'Hallelujah, we are not going to get far here' and do a new evaluation and come to the conclusion that has maybe more, uh, common sense. Not that the other person didn't have that, but he was in that frame of mind and thinking outside the box is not always easy …”
Q3	(Nurse) “… And that we are indeed allowed to have our opinion, but that we cannot decide and are not allowed to decide anything. And I can quite accept that, in most cases I can. To sometimes see afterwards like, ah look you see, we were right after all. …”
Q4	(Doctor) “… And because we know, a bit from experience, that if we are all on the same page, yes, then we are doing quite well. And if there is one person who really objects, we wait and see. Then time will tell what it will be, then that means it took another week or two weeks or something. If everyone is on the same page, things go faster, yes. And for that reason I think it is good that we have a relatively large group of doctors. And that there are different tendencies. Because I don't think it's very healthy if we all have the same nature. Because if you're too easy with that, yes, that's a self-fulfilling prophecy, huh. If you always say that some of them will not make it and you always stop, yes, then they will not make it, right … ”

If doctors and nurses did not actively consult each other, the participants pointed out the danger of persistent tunnel vision in each individual staff member. Staff members may then further build on their personal assessments of the care provided to a specific patient on the basis of their own observations, experiences and increasing frustrations or hopes (Table 5, Q2). A process of open and constructive communication in a team is required according to the participants, in order to broaden and nuance their own views, so that everyone can support univocal care goals. However, the data showed that such conversations more often resulted in mutual understanding for each others' opinions rather than in consensus about the type of care which is appropriate for a specific patient (Table 5, Q3). In the discussions with the healthcare team, uncertainties about the patient's wishes and future prospects were dominant. The interviews showed that the patient's therapy was often continued out of fear of making premature decisions (Table 5, Q4).

## 4 Discussion

Because a formal definition of appropriate care is lacking, this study attempts to increase understanding of the concept based on the perceptions of different clinical staff members, each of whom had their own role and responsibility in the patient's care process [24,25]. This can provide healthcare professionals with tools to apply appropriate care in daily practice.

Appropriate care does not appear to be an objective and self-contained concept, but takes shape around five elements. Initially there seemed to be consensus about the meanings and the contents of those elements. However, when the participants were asked to describe the elements by giving examples their meanings were no longer unambiguous. But, when afterwards comparing the framework with the literature, cited aspects, including “adequate care in accordance with the patient's wishes and in relation to future prospects and the patient's quality of life”, “interim evaluations of care goals” and “consensus regarding treatment decisions in the clinical team”, seem to correspond with descriptions of appropriate care in the current scientific literature [10,13,17,[26], [27], [28], [29], [30]].

The diversity of these perceptions in the patient cases was repeatedly the cause of fields of tension and frustrations among healthcare professionals. Nurses in particular seem to have confidence mainly in the competences of the team with regard to the delivery of care, but not so much in the decision-making process [31]. In order to achieve unity among healthcare professionals and to be able to support univocal appropriate care goals, healthcare professionals considered it appropriate to consult each other in time to clarify everyone's views [32,33]. The hierarchy among healthcare professionals, to which they often remained very faithful, was probably the only framework used in uncertain patient cases. The prognostic uncertainty, typically experienced by doctors with decision-making responsibility, often appears to dominate the decision-making process [25,31]. This can lead them to delay making decisions [34,35].

### 4.1 Theoretical implications

In order to promote interdisciplinary reflection on appropriate care, it seems desirable in the first instance that the broad elements of appropriate care are defined unambiguously. Although this is not common in the inductive approach to qualitative research, a conceptual framework was introduced based on what healthcare professionals considered potentially appropriate care. It seems useful to provide healthcare professionals with a unified terminology so that it can be discussed on a similar basis during staff consultations.

### 4.2 Practice implications

The current discussion on appropriate care no longer appears to address mainly the practical feasibility of therapeutic options, but rather their meaningfulness [[36], [37], [38]]. This is known in the literature as proportionate care [13,16,17,31]. Medical knowledge no longer plays a decisive role [25]. As a result, the participation of nurses in the decision-making process is equally valuable as that of doctors. In order to promote interdisciplinary reflection on appropriate care, a greater awareness of the complementarity of the views of doctors and nurses therefore seems desirable, as a second point of consideration. In order to better balance meaningful care and dignified care, it seems useful to integrate the views of doctors and nurses [39,40]. Moreover, it is expected that sharing and integrating the knowledge, experience and values of doctors and nurses can reduce uncertainty in decision-making processes [25]. It has already been demonstrated that prognostic estimates can be improved by, among other things, taking into account the perceptions of other team members, including nurses [16].

In addition, the newly introduced conceptual framework could serve as a guideline to address all elements included in the discussion of appropriate care.

### 4.3 Research implications

Additional studies can investigate whether the proposed conceptual framework can be verified and whether such a framework can actually facilitate the growth process of reflecting on appropriate care in an ICU team. Furthermore, it is likely that the results of this study are transferable to a Western non-ICU setting, nevertheless it could be interesting to study whether the framework is also applicable in countries with cultures that prioritise other values. In addition, little attention has been paid to the perspectives of the patients and their relatives in conceptualizing appropriate care.

### 4.4 Methodological limitations

The study has a number of limitations. The data reflect the perceptions and experiences of a small number of clinical staff members from three centers. Nevertheless, it was possible to achieve data saturation after fifteen interviews. Data from follow-up interviews no longer yielded new insights regarding the research question.

For the first interviews, participants were included based on their interest in participating in the study. To enhance the representativeness for the following interviews, participants were selected purposively. Contact persons addressed potential participants based on the criteria given by the researchers. However, the researchers did not have a clear view on personal preferences of contact persons. Furthermore, it is likely that cultural features such as religion, ethnicity, societal wellbeing, economic welfare and the national healthcare structure may have shaped the content of the interviews [41]. As a result, findings cannot be generalized automatically. Nevertheless it is likely that the results are transferable to non-ICU settings within the Western culture.

Finally, some of the participants knew the researcher, a nurse, which may have had an impact on the validity of the data. However, no direct colleagues of the researcher's own work environment were interviewed. In order to encourage participants to be truthful and open, the researcher maintained a respectful and confidential attitude at all times. The confidential and intimate atmosphere of an individual interview appeared to be desirable to collect in-depth valid data from the participants, given the sensitive and complex theme.

## 5 Conclusions

To describe appropriate care at ICU, doctors, doctors in training and nurses looked for aspects which can be included in a definition of appropriate care. It was difficult for healthcare professionals to find clarity about the type of care which was appropriate or not for the individual patient, for themselves and during communication in a team. In order to create clarity, a conceptual framework was proposed in this study to categorize their perceptions.

Healthcare professionals in the ICU seemed to find it important that the intensity of care is proportional to the future prospects that are acceptable to the patient (meaningful care). In addition, during the treatment process of the patient at ICU, the pursuit of these future perspectives must be proportional to the patient's suffering (dignified care). When high-quality care is provided in addition and social sustainability is taken into account, healthcare professionals use the term appropriate care. These elements of appropriate care are ideally evaluated in a team through a process of open and constructive communication, in which the perceptions of doctors and nurses can be complementary and are integrated.

## Ethical approval

This study received the approval of the Committee for Medical Ethics of the University Hospital Ghent (Belgian registration number: B670201734533).

## Participant consent

Each clinical staff member was informed about the purpose and procedure of the study via an information letter. Informed and voluntary consent to participate was obtained from each participant. The participants were informed of the possibility to withdraw from the study at any time. The confidentiality of the data and pseudonymity of the participants was guaranteed by the use of code numbers and the protection of data from third parties. No clinical staff members from the researcher's own work environment were interviewed.

## Data statement

The data is unsuitable for publication because the research data is confidential.

## Research reporting checklist

To promote the quality of the reporting of the research, the consolidated criteria for reporting qualitative research (COREQ) were consulted.

## Author contribution statement

Lore Huwel and Joke Van Eessen: Conceived and designed the experiments; Performed the experiments; Analyzed and interpreted the data; Contributed reagents, materials, analysis tools or data; Wrote the paper.

Jan Gunst, Manu L.N.G. Malbrain and Veerle Bosschem: Contributed reagents, materials, analysis tools or data; Wrote the paper.

Tom Vanacker: Conceived and designed the experiments; Analyzed and interpreted the data; Contributed reagents, materials, analysis tools or data.

Sofie Verhaeghe and Dominique D. Benoit: Conceived and designed the experiments; Analyzed and interpreted the data; Contributed reagents, materials, analysis tools or data; Wrote the paper.

## Funding statement

This research did not receive any specific grant from funding agencies in the public, commercial, or not-for-profit sectors.

## Data availability statement

The data that has been used is confidential.

## Declaration of interest's statement

The authors declare no competing interests.

## Additional information

No additional information is available for this paper.

## Footnotes

2 These quotes were originally spoken in (Belgian) Dutch by the participants. The written recordings have been translated using neural machine translation (NMT, viz. Google Translate) and post-edited (MTPE, machine translation, post-edited) by a language professional with an additional academic background in translation.

3 These quotes were originally spoken in (Belgian) Dutch by the participants. The written recordings have been translated using neural machine translation (NMT, viz. Google Translate) and post-edited (MTPE, machine translation, post-edited) by a language professional with an additional academic background in translation.

4 These quotes were originally spoken in (Belgian) Dutch by the participants. The written recordings have been translated using neural machine translation (NMT, viz. Google Translate) and post-edited (MTPE, machine translation, post-edited) by a language professional with an additional academic background in translation.
